# Stigma Associated with COVID-19 Among Health Care Workers in Indonesia

**DOI:** 10.1017/dmp.2021.93

**Published:** 2021-03-25

**Authors:** Amanda Yufika, Rovy Pratama, Samsul Anwar, Wira Winardi, Nurfanida Librianty, Nyoman Ananda Putri Prashanti, Tri Novita Wulan Sari, Prattama Santoso Utomo, Theresia Dwiamelia, Putu Pangestu Cendra Natha, Salwiyadi Salwiyadi, Febrivan Wahyu Asrizal, Ikram Ikram, Irma Wulandari, Sotianingsih Haryanto, Nice Fenobilire, Abram L Wagner, Kurnia Fitri Jamil, Mudatsir Mudatsir, Harapan Harapan

**Affiliations:** 1 Department of Family Medicine, School of Medicine, Universitas Syiah Kuala, Banda Aceh, Aceh, Indonesia; 2 Medical Research Unit, School of Medicine, Universitas Syiah Kuala, Banda Aceh, Aceh, Indonesia; 3 Department of Statistics, Faculty of Mathematics and Natural Sciences, Universitas Syiah Kuala, Banda Aceh, Aceh, Indonesia; 4 Department of Pulmonology and Respiratory Medicine, School of Medicine, Universitas Syiah Kuala, Banda Aceh, Aceh, Indonesia; 5 Department of Environmental Health, Faculty of Public Health, Universitas Indonesia, Depok, West Java, Indonesia; 6 Bangli Hospital, Bangli, Bali, Indonesia; 7 Sungai Dareh Hospital, Dharmasraya, West Sumatra, Indonesia; 8 Department of Medical Education and Bioethics, Faculty of Medicine, Public Health and Nursing, Universitas Gadjah Mada, Yogyakarta, Indonesia; 9 Panti Rahayu Hospital, Karangmojo, Yogyakarta, Indonesia; 10 Department of Internal Medicine, Faculty of Medicine, Universitas Udayana, Denpasar, Bali, Indonesia; 11 Department of Internal Medicine, Sanglah Hospital, Denpasar, Bali, Indonesia; 12 Department of Internal Medicine, School of Medicine, Universitas Syiah Kuala, Banda Aceh, Aceh, Indonesia; 13 Department of Internal Medicine, Dr. Zainoel Abidin Hospital, Banda Aceh, Aceh, Indonesia; 14 M Natsir Hospital, Solok, West Sumatra, Indonesia; 15 Dr H Yuliddin Away Hospital, Tapaktuan, Aceh, Indonesia; 16 M. Hatta Brain Hospital, Bukittinggi, West Sumatra, Indonesia; 17 Raden Mattaher Hospital, Jambi, Jambi, Indonesia; 18 Faculty of Medicine and Medical Sciences, Jambi University, Jambi, Jambi, Indonesia; 19 Pariaman Hostiptal, Pariaman, West Sumatra, Indonesia; 20 Department of Epidemiology, University of Michigan, Ann Arbor, Michigan, USA; 21 Tropical Disease Centre, School of Medicine, Universitas Syiah Kuala, Banda Aceh, Aceh, Indonesia; 22 Department of Microbiology, School of Medicine, Universitas Syiah Kuala, Banda Aceh, Aceh, Indonesia

**Keywords:** stigma, COVID-19, health care workers, indonesia

## Abstract

**Objective:**

The aim of this study was to assess the stigma associated with coronavirus disease - 2019 (COVID-19) among health care workers (HCWs) in Indonesia during the early phase of the pandemic.

**Methods:**

A cross-sectional study was conducted in 12 hospitals across the country in March, 2020. A logistic regression was employed to assess the association between stigma and explanatory variables.

**Results:**

In total, 288 HCWs were surveyed, of which 93.4% had never experienced any outbreaks. Approximately 21.9% of the respondents had stigma associated with COVID-19. HCWs who were doctors, had not participated in trainings related to COVID-19, worked in the capital of the province, worked at private hospitals, or worked at a hospital with COVID-19 triage protocols were likely to have no stigma associated with COVID-19.

**Conclusions:**

The stigma associated with COVID-19 is relatively high among HCWs in the early phase of the COVID-19 pandemic in Indonesia. Adequate dissemination of knowledge and adequate protection are necessary to reduce stigma among HCWs.

## Introduction

Coronavirus disease 2019 (COVID-19), caused by severe acute respiratory syndrome coronavirus 2 (SARS-CoV-2),^1^ was declared as a pandemic by the World Health Organization (WHO) on 11 March 2020 due to its alarming level of spread and severity.^2^ The emergence and spread of COVID-19 has caused confusion, anxiety and fear, and led to stigma on certain populations for being the reason for this outbreak.^3,4^ In the era of social media as it is today, myths and fake news around COVID-19 also spread rapidly, creating fear and stigma among the society.^5^ There was frequent use of terms like “Chinese virus” or “China virus” instead of COVID-19 on Twitter, indicating that stigma may be perpetuated on social media.^6^ A rise of stigma against people from China was also observed in Indonesia at the early phase of the pandemic, where the disease was referred to as “Chinese virus,” or as a punishment for Chinese suppression of Uighur Muslims.^7^

Stigma against particular ethnic groups was also reported in previous outbreaks.^8–11^ Fear, stigmatization, and discrimination towards Russian Jewish immigrants was reported in New York City during the typhoid and cholera outbreak in 1892.^10^ The Chinese-American community in San Francisco faced extreme discrimination during an outbreak of bubonic plaque in 1900, attributed to rats transported from Hong Kong.^8^ An outbreak of hantavirus in the United States in 1993 led to fear, stigmatization, and discrimination towards native Americans due to reports that referred the infection as a Navajo disease.^9^ During the severe acute respiratory syndrome (SARS) outbreak, stigma towards people who look Asian in the United States was reported, although the country was not severely affected.^8^ Stigma against health care workers (HCWs) also often occurs during outbreaks, due to their close contact with patients.^8,11^

Studies suggest that stigma is associated with negative health outcomes.^12–14^ Stigma and discrimination negatively affect public health efforts in diseases such as mental illness, epilepsy, tuberculosis, leprosy, and HIV/AIDS.^8,12,13^ Stigma caused people with HIV to hide their disease, avoid voluntary testing and counseling, and not seek for treatment, which pushed the epidemic underground. Fear of being stigmatized during an outbreak may cause people to deny clinical symptoms and not seek medical care.^5,8^ Furthermore, stigma may lead people to distrust the government, health professionals, and the health care system.^6,12,13^ In the context of the COVID-19 pandemic, public health measurements taken to contain the outbreak such as mask use, quarantine, and isolation fueled stigma towards the disease.^15^ It is therefore imperative that people trust their government and health care systems, so that they will be cooperative.^6^

Stigma associated with a particular disease is very dangerous, in particular if it comes from HCWs, as it may lead to poor health care service provision, and even denial of treatment to patients.^16^ According to previous studies, factors associated with stigma among HCWs include inadequate knowledge of the disease, irrational fear, working at an educational or public hospital, low level of education, and being male.^16–21^ Inadequate knowledge of transmission routes may lead to irrational fear and overestimated risk, which can be followed by stigma.^16,19^ As a newly emerging disease, knowledge about COVID-19 is still limited, thus people rely more on social media, where misconception and myths that create stigma often occur.^6,22^ With regard to gender, men showed more stigmatizing attitudes compared to women.^16^ Studies also showed that HCWs had higher perception of risk in others, as they feared getting infected with COVID-19,^23^ which may also lead to stigma.

The authors are aware of some studies exploring the stigma experienced by HCWs during the COVID-19 pandemic.^4,11^ There has however been no study exploring the views of HCWs themselves towards the disease. Stigma and other negative attitudes associated with the disease may harm public health measurements during pandemic like this. Therefore, this study was undertaken to assess the stigma associated with COVID-19 among HCWs in Indonesia.

## Methods

### Study design and setting

A cross-sectional study was conducted in 12 hospitals across Indonesia from March 6 to March 25, 2020 as part of Indonesia’s COVID-19 Project. Some results of this project have been published previously.^24–26^ The location of the hospitals was also taken into account, so that the 12 hospitals consisted of those located in urban and sub-urban areas.

### Study instrument

A questionnaire was developed to assess stigma associated with COVID-19 among HCWs. Information related to socio-demographic and workplace characteristics, HCW professional details, knowledge of COVID-19, exposure to COVID-19 information, and experience of outbreak-related trainings, was also collected. The questionnaire was tested among HCWs and was evaluated by 2 microbiologists before being used in the study.

### Data collection

HCWs in the 12 hospitals were approached and asked to participate in the study face-to-face. A brief overview of the study’s aims, risks, and benefits was provided by the research staff to the potential participants. HCWs who agreed to participate were asked to sign a written informed consent prior to the interview.

### Study variables

Stigma associated with COVID-19 among HCWs was assessed using a 6-item questionnaire. The questions used in the questionnaire were: (1) Chinese people are more prone to getting infected with SARS-CoV-2; (2) it is easier for SARS-CoV-2 to infect Chinese people compared to other ethnicities; (3) anyone who returned from China more than 14 days ago has to be avoided, although they show no symptoms of COVID-19; (4) it is fair for Europeans and Americans to suspect all Asian people visiting their countries are infected with COVID-19; (5) in my opinion, it is natural for a new disease to emerge in China due to their unusual eating habits; and (6) in my opinion, the COVID-19 outbreak is a curse towards Chinese people. Items were measured on a 5-point Likert type scale indicating respondents’ stance: 1 = Strongly agree, 2 = Agree, 3 = Not sure, 4 = Disagree, and 5 = Strongly disagree. The attitude scores for each participant were then summed up (ranging between 6 and 30), where lower scores indicated stigma. An 80% cut-off of the total score was used to indicate stigma in which participants who scored more than 80% were categorized as having no stigma.

Some explanatory variables were also collected: socio-demographic characteristics, characteristics of work and workplace, and knowledge of COVID-19. Socio-demographic characteristics included gender (male or female), age (≤ 30 years old or > 30 years old), and marital status (single or married). Characteristics of work included: (1) participants’ profession (doctor, nurse, or others); (2) the length of medical experience (in years); (3) involvement in management of any previous outbreak such as SARS, MERS, or Avian flu; (4) participation in any COVID-19 training courses; and (5) exposure to the latest information about COVID-19. Workplace characteristics included: (1) location of workplace (urban or sub-urban); (2) type of workplace (public or private hospital); (3) type of department (emergency room, intensive care unit (ICU), outpatient, infection, or others including laboratory and pharmacy); and (4) availability of COVID-19 protocol at working place. Knowledge of COVID-19 (transmission, symptoms, and prevention) was assessed using 13 questions, as used previously.^24^ Participants who scored more than 80% were classified as having good knowledge, while those who scored lower were classified as having poor knowledge.

### Statistical Analysis

Association between stigma and the explanatory variables were assessed using a 2-step logistic regression. Association between stigma and each explanatory variable was assessed separately in the first step. Only variables with *p* ≤ 0.25 in univariate analyses were included in the multivariate logistic regression. Analyses were conducted using SPSS ver. 17.0 (SPSS Inc., Chicago, IL, USA).

## Results

### Participants characteristics

In total, 288 HCWs participated in the study. Majority of them (65.3%) were women, more than half (59.7%) aged 30 or less and were married (59%). Most of them (93.4%) had never experienced any outbreaks, and had never participated in any COVID-19 related training courses (86.8%). Of the total, 51.7% of the participants had good knowledge (Table 1).

**Table 1.** Unadjusted and multivariable logistic regression analysis showing predictors of stigma associated with COVID-19 among healthcare workers (HCWs) in Indonesia (no stigma *vs.* stigma) (*n* = 288)

Variable	*n* (%)	No Stigma *n* (%)	Unadjusted		Multivariable	
			OR (95% CI)	*P*–value	OR (95% CI)	*P*–value
Gender						
Male (Reference group, R)	100 (34.7)	80 (80.0)	1			
Female	188 (65.3)	145 (77.1)	0.84 (0.46 – 1.53)	0.575		
Age group (year)						
30 or less (R)	172 (59.7)	136 (79.0)	1			
More than 30	116 (40.3)	89 (76.7)	0.87 (0.50 – 1.54)	0.637		
Marital status						
Single (R)	118 (41.0)	97 (82.2)	1		1	
Married	170 (59.0)	128 (75.3)	0.66 (0.37 – 1.19)	0.165	0.74 (0.31 – 1.76)	0.496
Healthcare professional group						
Doctor (R)	133 (46.2)	119 (89.5)	1		1	
Nurses	109 (37.8)	77 (70.6)	0.28 (0.14 – 0.57)	< 0.001	0.09 (0.03 – 0.25)	< 0.001
Others	46 (16.0)	29 (63.0)	0.20 (0.09 – 0.45)	< 0.001	0.19 (0.07 – 0.53)	0.002
Medical practice experience (years)						
Less than 5 years (R)	177 (61.5)	146 (82.5)	1		1	
5-10 years	54 (18.8)	36 (66.7)	0.43 (0.21 – 0.84)	0.014	0.80 (0.31 – 2.08)	0.644
More than 10 years	57 (19.8)	43 (75.4)	0.65 (0.32 – 1.34)	0.243	1.81 (0.57 – 5.75)	0.316
Experienced any outbreak prior to survey						
No (R)	269 (93.4)	208 (77.3)	1		1	
Yes	19 (6.6)	17 (89.5)	2.49 (0.56 – 11.09)	0.230	2.21 (0.34 – 14.54)	0.408
Have participated in any COVID-19-related training course						
No (R)	250 (86.8)	199 (79.6)	1		1	
Yes	38 (13.2)	26 (68.4)	0.56 (0.26 – 1.18)	0.124	0.19 (0.07 – 0.54)	0.002
Keep up to date on the latest information about case definitions for COVID-19						
No (R)	57 (19.8)	41 (71.9)	1		1	
Yes	231 (80.2)	184 (79.6)	1.53 (0.79 – 2.96)	0.209	0.76 (0.31 – 1.90)	0.562
Location of workplace						
Regency (R)	148 (51.4)	101 (68.2)	1		1	
Province	140 (48.6)	124 (88.6)	3.61 (1.93 – 6.74)	<0.001	3.12 (1.27 – 7.68)	0.013
Type of workplace						
Private hospital (R)	63 (21.9)	59 (93.6)	1		1	
Public hospital	225 (78.1)	166 (73.8)	0.19 (0.07 – 0.55)	0.002	0.05 (0.02 – 0.19)	< 0.001
Department						
Emergency department (R)	112 (38.9)	84 (75.0)	1		1	
ICU	18 (6.3)	12 (66.7)	0.67 (0.23 – 1.94)	0.457	0.95 (0.23 – 3.88)	0.940
Outpatient department	53 (18.4)	44 (83.0)	1.63 (0.71 – 3.76)	0.252	2.28 (0.75 – 6.91)	0.146
Infection department	37 (12.8)	36 (97.2)	12.00 (1.57 – 91.60)	0.017	8.97 (0.92 – 87.16)	0.059
Other departments including lab and pharmacy	68 (23.6)	49 (72.0)	0.86 (0.44 – 1.70)	0.663	0.44 (0.17 – 1.61)	0.097
The workplace has a protocol of triage and isolation for suspected COVID-19 patients						
No (R)	72 (25.0)	44 (61.1)	1		1	
Yes	216 (75.0)	181 (83.8)	3.29 (1.81 – 5.97)	< 0.001	3.47 (1.52 – 7.93)	0.003
Knowledge of COVID-19						
Poor (R)	139 (48.3)	100 (71.9)	1		1	
Good	149 (51.7)	125 (83.9)	2.03 (1.15 – 3.60)	0.015	1.00 (0.47 – 2.11)	0.994

### Level of Stigma and its Associated Factors

About 21.9% of the respondents had stigma associated with COVID-19. In univariate analysis, HCWs who were doctors, had practiced medicine for less than 5 years, worked at private hospitals, worked at the capital of the province, worked at hospitals with COVID-19 triage and isolation protocols, and had good knowledge of COVID-19 were likely to have no stigma associated with COVID-19 (Table 1). In multivariate analyses, doctors, those who had not participated in trainings related to COVID-19, who were working in the capital of the province, worked at private hospitals, or worked at hospitals with COVID-19 triage protocols, were likely to display no stigma associated with COVID-19 (Table 1).

Doctors were more likely to have no stigma associated with COVID-19 compared with nurses and other HCWs, with odds ratio (OR): 0.09 and 95% confidence interval (95% CI): 0.03 - 0.25), and OR: 0.19 (0.07 - 0.53), respectively. HCWs who had not participated in any COVID-19-related training courses were likely to have no stigma associated with the disease compared to those who had (OR: 0.19; 95% CI: 0.007 - 0.54). Participants who were working at provincial capitals (compared with those working at the regencies) and those who were working at private hospitals (compared with those working at public hospitals) were also likely to have no stigma associated with COVID-19 with OR: 0.05; 95% CI: 0.02 - 0.19, and OR: 0.05; 95% CI: 0.02-0.19, respectively. HCWs who were working at hospitals without COVID-19 triage protocols were 3.5 times likely to have stigma associated with COVID-19 (OR: 3.47; 95% CI: 1.52 - 7.93).

## Discussion

Our study found that 21.9% respondents had stigma associated with COVID-19. Taking into account that this study was conducted at the early phase of the COVID-19 outbreak in Indonesia, the stigmatizing attitudes might have been influenced by lack of information and knowledge about the disease. As mentioned in previous studies,^22,27^ knowledge, past experience, and beliefs can influence HCWs perceptions and attitudes toward a particular disease.

Our study found that doctors were more likely to have no stigma associated with COVID-19 compared to nurses and other HCWs. Having lower education was found to be associated with stigmatizing attitudes among HCWs, as also mentioned in previous studies.^16–18^ Nurses, midwives, or other HCWs in general had lower education compared to doctors, resulting in a poorer understanding of disease transmission, which internalized stigma. A significant knowledge gap regarding COVID-19 between doctors and other types of HCWs was also observed in another study.^22^ Moreover, nurses also have closer contact to the patients compared to doctors, which may lead to higher perceived risk and fear of being infected with COVID-19.^22^

Unexpectedly, this study found that HCWs who had never participated in any trainings related to COVID-19 were likely to have no stigma associated with COVID-19. This finding is interesting, as previous studies suggested that having better knowledge will diminish irrational fear, anxiety, and stigma and is associated with less stigmatized attitude.^22,27^ At the early phase of the pandemic, Indonesia experienced shortage of personal protective equipment (PPE) such as surgical masks, hazmat suits, and face shields.^28,29^ Some training related to COVID-19 was carried out during that phase. HCWs who had participated in the trainings might have higher perceived risk for knowing what should be done during the pandemic and the real situation they face, which results in more stigmatized attitude towards COVID-19.

We also noticed that HCWs who were working in private hospitals were less likely to have stigma when compared to those working in public hospitals. Most hospitals that prepared for COVID-19 in Indonesia were public; therefore, it might give the workers higher perceived risks of COVID-19, which could eventually lead to stigma. Furthermore, this study found that HCWs working at the capital of the province were 3 times more likely to have no stigma compared to those working in the sub-rural areas. This is understandable as living in the capital gives HCWs more access to information and training related to COVID-19, which contributed to having better knowledge.^23^ In addition, our study also found that HCWs whose working places had COVID-19 triage and isolation protocols for suspected patients were 3 times more likely to have no stigma compared to those whose workplaces had no clear protocols. Working in hospitals with clear protocols for COVID-19 patients might decrease the perceived risk of getting infected with SARS-CoV-2 among HCWs, resulting in less stigmatizing attitudes. Therefore, it is important to ensure that protective measurements and protocols are in place to decrease HSWs’ perceived risk of getting infected.

## Conclusion

A relatively high level of stigma associated with COVID-19 was observed among HCWs in Indonesia in the early phase of the pandemic. Being a doctor, having never participated in trainings related to COVID-19, working at the capital, working in a private hospital, and working at the hospital with COVID-19 triage protocols, made HCWs more likely to have no stigma associated with COVID-19. As the causes of stigma are multifactorial, the findings in this study need to be interpreted with caution. Nevertheless, strengthening dissemination of correct knowledge and information, as well as providing adequate protection for all HCWs, could be necessary for avoiding or reducing the stigma associated with COVID-19 among HCWs.
